# *Artemisia* *herba alba* Outperforms Indomethacin with Multitarget Efficacy and Safety in CFA Arthritic Model

**DOI:** 10.3390/antiox15020190

**Published:** 2026-02-02

**Authors:** Hicham Wahnou, Martin Ndayambaje, Imane Nait Irahal, Zaynab Ouadghiri, Wafaa Taha, Asmaa Mazti, Riad El Kebbaj, Youness Limami, Mounia Oudghiri

**Affiliations:** 1 Laboratory of Integrative Biology, Faculty of Sciences Ain Chock, Hassan II University, Casablanca 20100, Morocco; ndayambajemartin12@gmail.com (M.N.); zaynabouadghiri1@gmail.com (Z.O.); tahawafaa37@gmail.com (W.T.); mouniaoudghiri@gmail.com (M.O.); 2 Sciences and Engineering of Biomedicals, Biophysics and Health Laboratory, Higher Institute of Health Sciences, Hassan First University, Settat 26000, Morocco; elkebbajriad@gmail.com (R.E.K.); youness.limami@gmail.com (Y.L.); 3 Laboratoire Santé, Environnement et Biotechnologie (LSEB), Faculté des Sciences Ain Chock, Université Hassan II de Casablanca, BP5366 Maarif, Casablanca 20100, Morocco; imanenaitirahal@gmail.com; 4 Laboratory of Pathological Anatomy, Ibn Rochd University Hospital-Casablanca, Casablanca 20100, Morocco; maztiasmae@gmail.com; 5 Mohammed VI Center for Research & Innovation (CM6RI), Rabat 10112, Morocco

**Keywords:** monoarthritis, anti-inflammatory, antioxidant, behavioral assessment, natural therapeutics, toxicity, non-steroidal anti-inflammatory drugs

## Abstract

Rheumatoid arthritis remains a major clinical challenge requiring safer and more effective alternatives to conventional non-steroidal anti-inflammatory drugs (NSAIDs). This pioneering study evaluated the anti-inflammatory, analgesic, antioxidant, and safety effects of *Artemisia herba alba* extract in complete Freund’s adjuvant (CFA)-induced arthritis in rats. Animals received oral *Artemisia herba alba* (250 or 500 mg/kg), indomethacin (3 mg/kg), or saline for 15 days. CFA induced marked joint inflammation, mechanical allodynia, locomotor impairment, and oxidative stress. Treatment with *Artemisia herba alba* 500 mg/kg significantly reduced paw swelling, improved mobility in the open-field test, and markedly attenuated pain hypersensitivity. In parallel, biochemical analyses showed restoration of total antioxidant capacity, prevention of lipid peroxidation, and normalization of creatinine levels. Unlike indomethacin, which induced hepatotoxicity (elevated ASAT (Aspartate Aminotransferase)/ALAT (Alanine Aminotransferase)) and pronounced oxidative stress, *Artemisia herba alba* preserved liver and kidney function and did not produce histopathological alterations. Histological findings further indicated reduced inflammatory infiltrate and cartilage protection, particularly at 500 mg/kg. Taken together, these results suggest that *Artemisia herba alba* displays a multitarget effect with anti-inflammatory, antioxidant and analgesic activity, along with a superior safety profile compared with indomethacin, consistent with reports from other phenolic-rich natural products. However, findings should be interpreted in light of the small sample size and preclinical study design, and further mechanistic and clinical investigations are warranted.

## 1. Introduction

Arthritis encompasses a heterogeneous group of chronic inflammatory disorders characterized by persistent synovial inflammation, progressive cartilage degradation, and joint destruction [1]. The resulting symptoms, pain, stiffness, swelling, and loss of function, profoundly impact daily activities and reduce quality of life [2]. Globally, arthritis affects over 350 million people and remains a leading cause of disability [2,3]. Rheumatoid arthritis, one of the most severe forms, is associated with systemic inflammation, immune dysregulation, and oxidative damage [4]. Despite advances in biologic therapies and synthetic disease-modifying antirheumatic drugs (DMARDs), many patients experience incomplete symptom relief or develop adverse drug reactions [5]. Consequently, the search for alternative, more tolerable treatments is ongoing and urgent.

Preclinical models have become indispensable for evaluating potential therapies in arthritis. The complete Freund’s adjuvant (CFA)-induced arthritis model in rodents is widely employed due to its reproducibility and similarity to human monoarthritis [6]. A single subcutaneous injection of CFA elicits a localized and sustained inflammatory response, characterized by synovial hyperplasia, immune cell infiltration, nociceptive hypersensitivity, oxidative stress, and progressive joint damage [6,7]. This model provides a robust platform for simultaneously assessing anti-inflammatory, analgesic, antioxidant, and tissue-protective effects of candidate compounds. It also allows researchers to evaluate potential side effects on systemic physiology, including hepatic and renal function.

Nonsteroidal anti-inflammatory drugs (NSAIDs) such as indomethacin are commonly prescribed to manage arthritis-related pain and inflammation [8]. However, long-term NSAID use is associated with significant gastrointestinal, renal, and hepatic toxicity, as well as oxidative stress and impaired mitochondrial function [9]. These concerns have driven interest in natural therapeutic alternatives that may offer comparable efficacy with improved safety. Medicinal plants, long employed in traditional systems, are now recognized as valuable sources of bioactive secondary metabolites with anti-inflammatory, analgesic, and antioxidant properties [10].

*Artemisia herba alba* (white wormwood), a perennial shrub native to arid and semi-arid regions of North Africa, the Middle East, and the Mediterranean basin, holds a prominent place in traditional medicine [11]. In Moroccan ethnobotany, *Artemisia herba alba* is used to treat inflammatory diseases, joint pain, gastrointestinal disorders, and infections [11]. Phytochemical studies have identified a rich diversity of secondary metabolites in the plant, including flavonoids (quercetin, luteolin), phenolic acids (caffeic, ferulic, and chlorogenic acids), sesquiterpene lactones, and essential oils, many of which exhibit potent antioxidant, antimicrobial, and anti-inflammatory activities [12,13]. While several studies have reported in vitro effects or traditional uses of *Artemisia herba alba* [12,13,14], to date, no studies have thoroughly assessed its antioxidant, anti-inflammatory and analgesic actions in validated in vivo models of arthritis, nor have they systematically evaluated its safety profile.

In this context, the present study investigates the therapeutic potential of *Artemisia herba alba* hydroethanolic extract in a rat model of CFA-induced monoarthritis. We aimed to assess its effectiveness in attenuating joint inflammation and pain while evaluating its systemic safety in comparison to indomethacin, a standard anti-inflammatory drug.

## 2. Materials and Methods

### 2.1. Collection and Preparation of Artemisia herba alba Extract

Samples of *Artemisia herba alba* were collected in late February 2024 within Morocco’s Souss-Massa-Drâa region (coordinates: 29°39′30.5″ N, 9°06′30.0″ W), at the first appearance of early buds. Taxonomic identification was confirmed by experts at the Scientific Institute of Rabat, and a preserved voucher specimen (herbarium accession code: RAB11175) was deposited for future reference.

The aerial parts of the plant were selected for analysis. Post-harvest, the plant material was dried naturally in a shaded, well-ventilated area to prevent degradation. The desiccated biomass was then pulverized into a homogeneous powder, stored in light-protected containers, and kept under stable conditions until required for experimentation. The hydroethanolic (EtOH-H_2_O 70%) extract of the aerial parts was prepared as previously described [12,13].

### 2.2. Animals

Adult male *Wistar* rats were obtained from the animal research facility of Hassan II University’s Laboratory of Immunology and Biodiversity. Following acquisition, the animals underwent a 20-day acclimatization period in controlled environmental conditions, maintained at 24 ± 5 °C with 55 ± 10% relative humidity under a standardized 12 h light/dark cycle (lights on 08:00–20:00). Throughout the study, rats had free access to standard laboratory chow and drinking water. Prior to behavioral testing, animals were familiarized with the experimental environment. This study was designed and reported following the ARRIVE guidelines for preclinical research and was approved by Local Ethics Committee of Faculty of Sciences, Rabat, Morocco (Project: CEFSR/PR07).

### 2.3. CFA-Induced Arthritis

Experimental mono-arthritis was induced in male *Wistar* rats following the protocol outlined by Shirani et al. [15] with some modifications. Briefly, four groups with four animals in each (n = 16) received a single intra-articular injection of 0.1 mL CFA into the left hind footpad. The control group (n = 4) received no injection. Ketamine (20 mg/kg b.w., i.p.) was used for anesthesia during the induction process.

**Figure 1:**
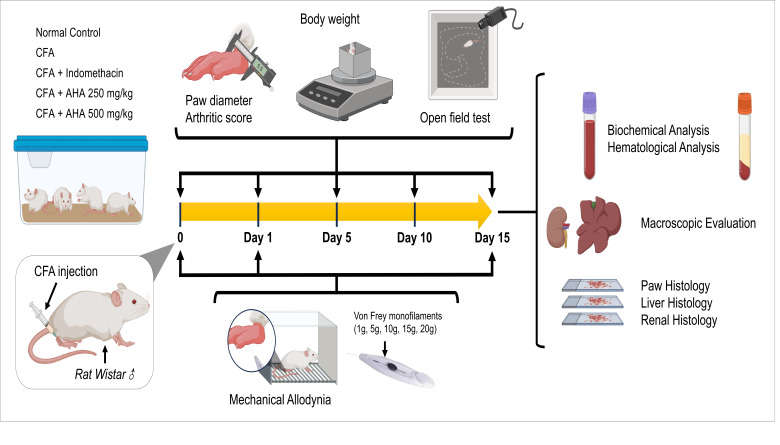
Experimental design of CFA-induced arthritis in male (♂) *Wistar* and the evaluation of different parameters.

The CFA-treated rats were assigned specific treatments over the 15-day period. Group I was treated orally with 3 mg/kg of indomethacin, serving as an anti-inflammatory reference drug, Group II and Group III were treated orally with *Artemisia herba alba* EtOH-H_2_O extract at a dosage of 250 mg/kg and 500 mg/kg, respectively, while Group IV was treated orally with saline (vehicle). The doses used in this study are selected based on a previous study showing effective anti-inflammatory potential using the same extract [13]. Oral treatments were administered one hour prior to arthritis induction on day 0, and one hour before testing on the other study days. On day 15, rats were humanely sacrificed, and the ankle joints were cleaned using 0.9% NaCl solution and placed in buffered formalin 10% for histopathological analysis. In evaluating arthritis, paw diameters were assessed at specified intervals using an electronic vernier caliper throughout the experimental period, see Figure 1. The paw diameter *change* (%) was determined by the following formula:

(1) $$Change\;\left(\%\right)=\left(\frac{\mathrm{dt}-d0}{d0}\right)\times 100$$

where “dt” signifies the paw measured on days (5, 10 or 15) and “d0” represents the paw diameter on day 0.

The *inhibition* % on the other hand was determined by the formula

(2) $$Inhibition\;\left(\%\right)=\left(1-\frac{Dt}{Dc}\right)\times 100$$

where “*Dt*” is the difference in paw diameter in the drug-treated groups and “*Dc*” represents the difference in paw diameter in CFA arthritic group.

Arthritic severity was semi-quantified using a standardized clinical scoring system adapted from Vijayalaxmi et al. [16], in which the arthritic index for each limb is assessed by macroscopic examination of inflammation, erythema, redness, and swelling. Each limb is visually scored on an ordinal scale from 0 to 5, where 0 indicates no observable changes, 1–4 represent increasing severity of swelling and redness, and 5 corresponds to severe joint deformity with complete immobility of the limb (Table 1).

### 2.4. Open Field Test

To assess the mobility, and anxiety-like behavior in rats, the open field (OF) test was performed in an opaque plastic arena (50 × 50 × 50 cm), scaled appropriately for rats. During the test phase, each rat was placed in the center of the arena and allowed to explore freely for 15 min [17], Figure 1. Behavior was recorded using an overhead digital camera, and the total distance traveled (to assess general locomotor activity) was tracked using the open-source multi-platform application (AnimAppVersion 0.1.5.6) [18].

The arena was thoroughly cleaned with 75% ethanol between trials to remove residual odor cues. Rats displaying thigmotaxis (preferential movement along walls) were considered more anxious, whereas increased central zone exploration indicated lower anxiety-like behavior.

### 2.5. Mechanical Allodynia: Von Frey Filament Testing

Semmes–Weinstein monofilaments (1 g, 5 g, 10 g, 15 g, 20 g bending force) were used to assess mechanical hypersensitivity following previous studies [19]. In a normal, non-inflamed state, a light filament is typically ignored, while a stronger filament may elicit a brief, reflexive withdrawal. Following an injury or inflammatory insult like CFA, this threshold often lowers, a condition known as tactile allodynia, where even light, normally innocuous touch is perceived as painful and elicits a sharp withdrawal [20].

Rats were acclimated to a mesh platform (20 × 20 cm) for 30 min/day over 3 days prior to testing. On days 0 (post-CFA), 1, and 15 post-CFA induction, filaments were applied in ascending order (1 g to 20 g) to the plantar hind paw five times each, with 5 s intervals between applications, Figure 1. A sharp paw withdrawal was recorded as a positive response. The % *withdrawal response* was determined using Dixon’s up–down method to quantify mechanical sensitivity.

(3) $$\%\;Withdrawal\;Response=\frac{\mathrm{Wpos}}{\mathrm{Ntotal}}\times 100$$

with “Wpos” denoting the number of positive paw withdrawals during testing, and “Ntotal” representing the total applications per filament (5 applications per rat).

### 2.6. Hematological Analysis

At the end of the experimental protocol (Day 15), the animals were humanely sacrificed under deep anesthesia induced by an intraperitoneal injection of ketamine (100 mg/kg) [21].

Following confirmation of deep anesthesia, cardiac puncture was performed to collect whole blood samples using sterile syringes pre-coated with EDTA as an anticoagulant. The blood samples were gently homogenized and processed immediately to prevent cell degradation. In addition, a second blood sample was collected in a dry tube (without anticoagulant) and allowed to clot at room temperature for 30 min before centrifugation at 3000 rpm for 10 min. The resulting serum was carefully separated and stored at −20 °C until biochemical analyses were performed.

Hematological parameters were assessed using a fully automated hematology analyzer (the Sysmex XN1000; Sysmex Corporation, Kobe, Japan)). The analysis included red blood cells (RBC), neutrophils, lymphocytes, monocytes, basophils, and platelets.

### 2.7. Serum Antioxidant Capacity

The serum total antioxidant capacity (TAC) and free radical scavenging activity were assessed using two spectrophotometric assays in a 1 mL final volume. First, TAC was determined via the phosphomolybdenum (molybdate reduction) assay [22]. Briefly, 50 µL of undiluted serum was reacted with 950 µL of fresh assay reagent (0.6 M sulfuric acid, 28 mM sodium phosphate, and 4 mM ammonium molybdate mixed 1:1:1, *v*/*v*/*v*) in a 1.5 mL tube. After incubation at 95 °C for 90 min and cooling, the absorbance of the resulting green phosphomolybdenum (V) complex was measured at 695 nm against a reagent blank. Second, the radical scavenging activity was evaluated using the 2,2-diphenyl-1-picrylhydrazyl (DPPH^•^) assay [23]. A 125 µL aliquot of serum diluted 1:5 (*v*/*v*) with PBS (pH 7.4) was mixed with 875 µL of a 0.1 mM methanolic DPPH^•^ solution. Following a 30 min incubation in the dark, the absorbance was measured at 517 nm.

(4) $$\%\;DPPH\;Inhibition=\frac{(\mathrm{Ab}\;-\left(\mathrm{As}\;-\;\mathrm{Ac}\right)}{\mathrm{Ab}}\times 100$$

where Ab is the absorbance of the DPPH^•^ blank, As is the absorbance of the serum reaction mixture with DPPH^•^, and Ac is the absorbance of the serum sample control.

### 2.8. Biochemical Analysis

The protein concentration in the samples was determined using the Bradford method with bovine serum albumin as the standard [24]. Lipid peroxidation was assessed by measuring thiobarbituric acid reactive substances (TBARS), particularly malondialdehyde (MDA), following the protocol of De León et al. [25]; this involved reacting the samples with thiobarbituric and trichloroacetic acids under acidic conditions, heating at 100 °C, and measuring absorbance at 535 nm. Catalase (CAT) activity was evaluated based on the decomposition of hydrogen peroxide (H_2_O_2_), monitored at 240 nm as described by Aebi et al. [26], and expressed as µmol H_2_O_2_ consumed per minute per mg protein, using an extinction coefficient of 39.4 µM^−1^ cm^−1^. Succinate dehydrogenase (SDH) activity was determined according to King et al. [27] by measuring the reduction in dichlorophenolindophenol (DCIP) at 625 nm, with results expressed as µmol DCIP reduced per minute per mg protein, using an extinction coefficient of 19,100 M^−1^ cm^−1^. In addition, serum biochemical parameters including aspartate aminotransferase (ASAT), alanine aminotransferase (ALAT), urea, and creatinine were quantified using an automated biochemical analyzer (Biolis24i; Tokyo Boeki Medisys Inc., Tokyo, Japan) according to standard enzymatic methods.

### 2.9. Organ Weight and Indexing

The liver and both kidneys (right and left) were carefully rinsed with a 0.9% sodium chloride solution. Following a macroscopic inspection, the organs were weighed. The organ index (*OI*) was subsequently calculated using the formula

(5) $$OI=\frac{\mathrm{Wo}}{\mathrm{Wb}}\times 100$$

where *Wo* denotes the weight of the specific organ and *Wb* corresponds to the total body weight of the animal.

### 2.10. Histopathological Analysis

Rats’ left tibiotarsal (ankle) joints, were initially decalcified in 10% EDTA at 4 °C for 30 days, whereas liver, and kidneys were directly fixed in 10% buffered formalin at 4 °C. Following fixation, the samples underwent dehydration using a gradient of ethanol solutions with varying concentrations (70%, 80%, 90%, 95%, and 100%). Subsequently, the samples underwent two changes of xylene for clarification. They were then impregnated with liquid paraffin, embedded, and sectioned. Sections of 4–5 μm thickness were prepared and stained with hematoxylin and eosin (H&E) staining for histological examination. The histopathological examination was performed by a board-certified pathologist (AM). Semi-quantitative histopathological scoring of joint sections on inflammatory cell infiltration and bone erosion were blindly assessed by a pathologist and assigned scores of 0–4 as previously described [28,29,30].

### 2.11. Statistical Analysis

Statistical analysis was performed using GraphPad Prism 9.4.0 software (GraphPad Software Inc., San Diego, CA, USA). One-way and two-way ANOVA tests were utilized to analyze multiple groups followed by Tukey’s multiple comparisons test. Differences were considered statistically significant at *p* < 0.05.

## 3. Results

### 3.1. Effect on Paw Edema and Body Weight

**Figure 2:**
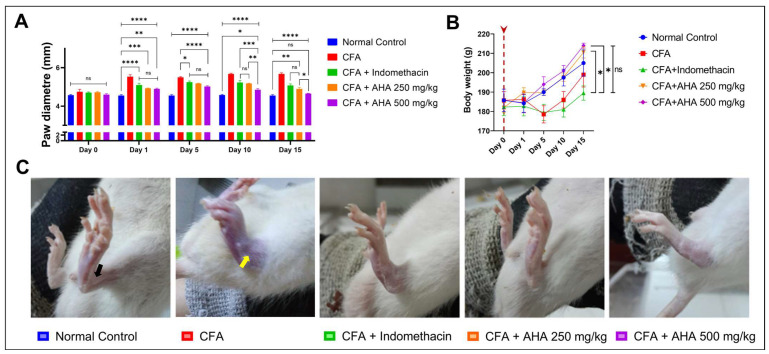
*Wistar* rats were immunized with an injection CFA on day 0. (**A**) Represents the paw diameter changes over the 15 experimental days, (**B**) Measurement of body weight in grams. All measurements were made one day after injection and every five days as described in the Methods Section, red arrows represent days of injections; and (**C**) the representative pictures of rats left hind paw swelling tibiotarsal joints (yellow arow), and normal tibiotarsal joint (black arow); data are mean ± SEM of four rats. Multiple comparisons between groups represented are, ns: non-significant, * *p* < 0.05, ** *p* < 0.01, *** *p* < 0.001, **** *p* < 0.0001.

In the normal control group, paw diameter remained stable throughout the experiment, with no significant changes observed compared to day 0. Conversely, the arthritic control group (CFA) exhibited a progressive increase in paw diameter, reaching 5.675 ± 0.06 mm by day 15, indicative of inflammation and disease progression (*p* < 0.0001). Treatment with indomethacin resulted in a significant reduction in paw diameter compared to the arthritic control group 5075 ± 0.08 mm by day 15 (*p* < 0.01). Rats treated with indomethacin showed a significant reduction in paw diameter at day 15, reaching 5075 ± 0.08 (*p* < 0.001). On the other hand, *Artemisia herba alba* treatment at 250 mg/kg displayed a significant decrease in paw diameter compared to the arthritic control group, with a measurement of 4.9 ± 0.07 mm by day 15. Notably, treatment with *Artemisia herba alba* at 500 mg/kg led to a significant reduction in paw diameter particularly evident from day 5 onwards (*p* < 0.001), reaching 4.65 ± 0.02 mm by day 15, indicating a pronounced inhibition of inflammation compared to the arthritic control group, see Figure 2A, and Table S1.

Transitioning to the body weight variation, rats in the normal control group exhibited a gradual increase in body weight over the course of the experiment, with mean body weight rising from 185.75 ± 5.8 g on day 0 to 205 ± 6.78 g by day 15, indicative of normal growth and development. In contrast, rats in the CFA-induced arthritis group showed fluctuations in body weight, with no statistically significant changes compared to day 0. The mean body weight ranged from 185.75 ± 14.49 g on day 0 to 178.5 ± 4.44 by day 5, increasing to 199 ± 6.37 g by day 15. Treatment with indomethacin did not significantly alter body weight compared to day 0, with mean body weight ranging from 182.25 ± 4.46 g on day 0 to 189.5 ± 3.7 g by day 15. Similarly, rats treated with *Artemisia herba alba* at both 250 mg/kg and 500 mg/kg exhibited a pattern of weight gain similar to the normal control group, suggesting that the administration of *Artemisia herba alba* did not negatively impact overall weight change. The mean body weight ranged from 182 ± 2.55 g to 214 ± 1.29 g for the *Artemisia herba alba* 250 mg/kg group and from 182 ± 2.55 g to 210.5 ± 2.9 g for the *Artemisia herba alba* 500 mg/kg group over the duration of the experiment, see Figure 2C and Tables S1 and S2.

### 3.2. Von Frey Filament Testing and Arthritis Severity Progression

**Figure 3:**
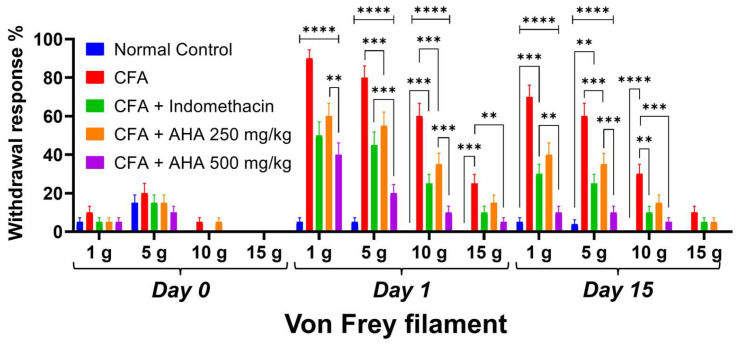
Mechanical withdrawal response percentages (%) for von Frey filament tests (1 g, 5 g, 10 g, 15 g) across experimental groups (normal control, CFA, CFA + indomethacin, CFA + *Artemisia herba alba* (AHA) at 250 mg/kg, and 500 mg/kg) at days 0, 1, and 15 post-CFA induction. Data are mean ± SEM of four rats. Multiple comparisons between groups are represented. ns: non-significant, ** *p* < 0.01, *** *p* < 0.001, **** *p* < 0.0001.

**Figure 4:**
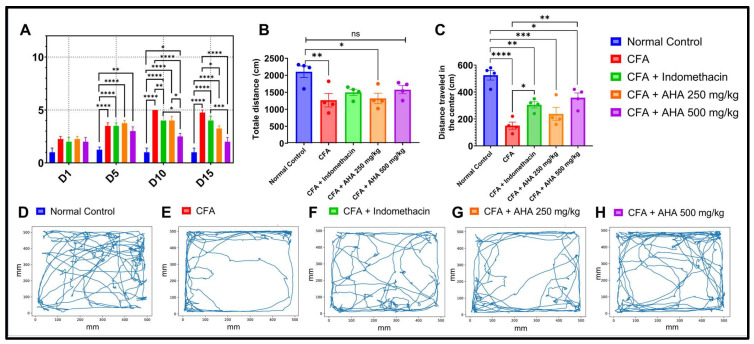
(**A**). Arthritis severity progression across groups over time, with joint inflammation scores assessed every 5 days using a standardized visual scoring system. (**B**) Total distance traveled (cm) in the open field test during 10 min. (**C**) Distance traveled in the center zone (cm), reflecting anxiety-like behavior. (**D**–**H**) Representative movement trajectories for each group: (**D**) normal control (healthy baseline), (**E**) CFA (untreated disease model), (**F**) CFA + indomethacin (standard treatment), (**G**) CFA + *Artemisia herba alba* 250 mg/kg, and (**H**) CFA + *Artemisia herba alba* 500 mg/kg. Data are mean ± SEM of four rats. Multiple comparisons between groups are represented. ns: non-significant, * *p* < 0.05, ** *p* < 0.01, *** *p* < 0.001, **** *p* < 0.0001.

Von Frey filament testing demonstrated that CFA-induced arthritic rats developed significant mechanical hypersensitivity by day 1, exhibiting 90 ± 4.5% and 80 ± 6.1% withdrawal responses to 1 g and 5 g filaments, respectively (*p* < 0.0001 vs. normal controls), which persisted through day 15 (70 ± 6.1% and 50 ± 6.7%). Treatment with *Artemisia herba alba* 500 mg/kg showed superior efficacy, reducing day 1 responses to 40 ± 6.1% (*p* < 0.0001 vs. arthritic controls) and normalizing thresholds by day 15 (10 ± 3.2%, *p* < 0.0001), while *Artemisia herba alba* 250 mg/kg and indomethacin also provided significant protection (day 5; 10 g: 60 ± 6.7% and 50 ± 7.1%, *p* < 0.001), a clear dose–response relationship was observed, with *Artemisia herba alba* 500 mg/kg significantly outperforming the 250 g/kg dose (day 1; 10 g responses: 10 ± 3.2% vs. 35 ± 5.7%, *p* < 0.001), while normal controls maintained minimal responses throughout the study Figure 3 and Table S3. These results were confirmed by the clinical score severity, as shown in Figure 4A. In fact, the CFA control group exhibited an elevated score of 5 (3 out of 4 rats), reflecting joint swelling and immobility of the limb Figure 2C. In contrast, the group treated with *Artemisia herba alba* 500 mg/kg showed a significant reduction in clinical score, bringing it to a level comparable to the normal control. The groups treated with *Artemisia herba alba* 250 mg/kg and indomethacin displayed intermediate scores, demonstrating a partial but significant reduction in disease severity.

### 3.3. Open Field Analysis

The results demonstrate a significant impact of arthritis induced on the locomotor and exploratory behaviors in rats, see Figure 4. The normal control group exhibited the highest total distance traveled (2104 ± 337.79 cm) and center zone exploration (525 ± 71.88 cm), indicative of normal mobility and low anxiety. In contrast, CFA-arthritic rats showed a marked reduction in total locomotor activity (1265.5 ± 395.1 cm, *p* < 0.01) and a drastic decline in center zone activity (150 ± 53.54 cm, *p* < 0.0001), reflecting pain-induced immobility and heightened anxiety. Treatment with indomethacin significantly improved center zone exploration (305 ± 46.55 cm, *p* < 0.05), confirming its anti-inflammatory and anxiolytic efficacy. *Artemisia herba alba* at 500 mg/kg also showed notable improvements in center zone activity (357.5 ± 71.36 cm, *p* < 0.01) with no significant difference from the normal control group. On the other hand, the lower dose of *Artemisia herba alba* (250 mg/kg) produced a non-significant change in total locomotion, (1318 ± 307.98 cm) and center exploration (237.5 ± 97.43 cm) in comparison with the CFA group. These findings suggest that *Artemisia herba alba*, particularly at higher doses, may exert beneficial effects by alleviating inflammation-related motor deficits and anxiety-like behaviors, with effects comparable to those of the reference drug indomethacin.

### 3.4. Serum Analysis

#### 3.4.1. Total Antioxidant Capacity (TAC) and DPPH

**Figure 5:**
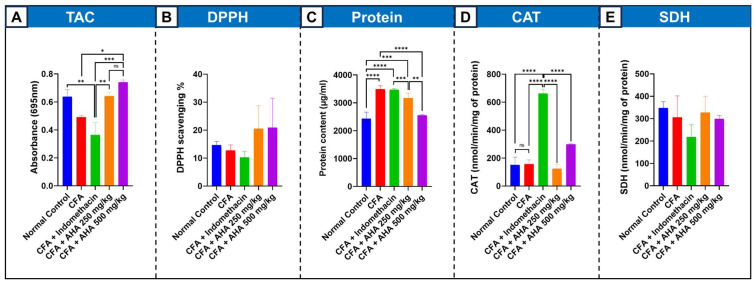
Effects of *Artemisia herba alba* (AHA) extract on oxidative stress and antioxidant-related parameters in CFA-induced arthritic rats. (**A**) total antioxidant capacity (TAC) expressed as absorbance at 695 nm. (**B**) Free radical scavenging activity measured by the DPPH assay (% inhibition). (**C**) Total protein content (µg/mL). (**D**) Catalase (CAT) activity (nmol/min/mg protein). (**E**) Succinate dehydrogenase (SDH) activity (nmol/min/mg protein). Data are presented as mean ± SEM. Statistical significance was determined by one-way ANOVA followed by appropriate post hoc tests. * *p* < 0.05, ** *p* < 0.01, *** *p* < 0.001, **** *p* < 0.0001.

In the CFA-induced arthritic group, TAC levels showed no significant change compared to the normal control group (*p* > 0.05). However, treatments of the induced arthritis model significantly depleted the systemic antioxidant capacity, as measured by TAC. Treatment with the standard drug indomethacin significantly reduced TAC (*p* < 0.01), remaining low at 0.364 nm. In contrast, *Artemisia herba alba* extract demonstrated a potent, dose-dependent antioxidant restorative effect. The lower dose of 250 mg/kg restored TAC to normal levels at 0.642 nm. On the other hand, the higher dose of 500 mg/kg restored TAC to 0.741 nm and was 16% higher than the normal baseline Figure 5A. In parallel, the evaluation of serum antioxidant capacity using the DPPH free radical scavenging assay revealed no statistically significant differences between the experimental groups, although a slight increase in % DPPH scavenging was observed in *Artemisia herba alba*-treated groups Figure 5B.

#### 3.4.2. Protein Content

Protein content (µg/mL). CFA-induced arthritis led to a significant increase in total protein levels compared with the normal control group (2428.95 ± 136.40 µg/mL), reaching 3491.23 ± 70.18 µg/mL (*p* < 0.0001). Treatment with the reference drug indomethacin did not significantly reverse this elevation, as protein levels remained comparably high (3469.30 ± 22.81 µg/mL; non-significant vs. arthritic control). In contrast, administration of *Artemisia herba alba* extract induced a dose-dependent and statistically significant reduction in protein content. The 500 mg/kg dose significantly decreased protein levels to 3166.67 ± 103.42 µg/mL (*p* < 0.0001 vs. arthritic control) with values approaching those of the normal control group, while the 250 mg/kg dose produced no significant difference in comparison to arthritic group reaching 2550.88 ± 15.29 µg/mL

#### 3.4.3. Catalase (CAT) Activity

The baseline activity of the key antioxidant enzyme CAT was similarly low in both normal and arthritic control groups, at approximately 150 nmol/min/mg of protein. A dramatic divergence was observed following drug treatments. Indomethacin triggered an over four-fold increase in CAT activity to 661.35 nmol/min/mg protein, suggesting a strong compensatory upregulation likely in response to drug-induced oxidative stress. Conversely, *Artemisia herba alba* extract did not significantly alter CAT activity at either the 250 mg/kg or 500 mg/kg doses, with values remaining near the baseline levels at approximately 120–125 nmol/min/mg protein, Figure 5D.

#### 3.4.4. Succinate Dehydrogenase (SDH) Activity

The activity of the mitochondrial enzyme SDH, a marker for cellular energy metabolism, remained stable across all experimental groups. Neither the induction of arthritis nor treatment with indomethacin or *Artemisia herba alba* extract at either dose produced a statistically significant change. All values fluctuated around a mean of 300 nmol/min/mg protein, indicating that the fundamental mitochondrial energy function was preserved under all conditions in this study, see Figure 5E.

#### 3.4.5. Malondialdehyde (MDA)

**Figure 6:**
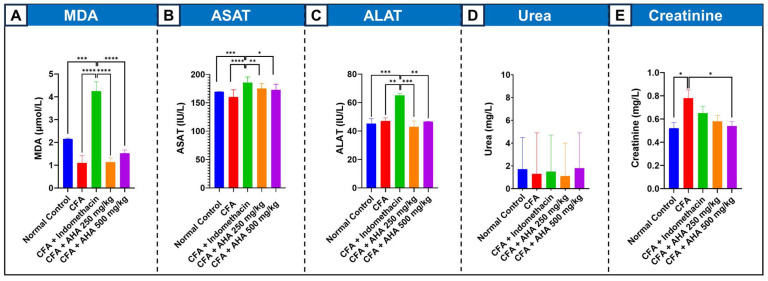
Effects of *Artemisia herba alba* (AHA) extract on lipid peroxidation and hepatorenal biochemical markers in CFA-induced arthritic rats. (**A**) Malondialdehyde (MDA) levels (µmol/L) as an index of lipid peroxidation. (**B**) Aspartate aminotransferase (ASAT) activity (U/L). (**C**) Alanine aminotransferase (ALAT) activity (U/L). (**D**) Serum urea levels (mg/L). (**E**) Serum creatinine levels (mg/L). Data are presented as mean ± SEM. Statistical significance was determined by one-way ANOVA followed by appropriate post hoc tests. * *p* < 0.05, ** *p* < 0.01, *** *p* < 0.001, **** *p* < 0.0001.

The analysis of MDA, a marker of lipid peroxidation and oxidative damage, showed no significant difference between the normal control and CFA-arthritic group. The drug treatments, on the other hand, yielded starkly different outcomes. Indomethacin caused severe lipid peroxidation, elevating MDA levels to 4.25 µmol/L—nearly four times higher than the arthritic control. In contrast, both doses of *Artemisia herba alba* extract maintained MDA at low levels (1.13 and 1.52 µmol/L), Figure 6A.

#### 3.4.6. Liver Function Enzymes (ASAT and ALAT)

The induction of arthritis itself did not cause significant liver function change in ASAT and ALAT in comparison to normal control. However, indomethacin treatment caused clear drug-induced hepatotoxicity, significantly elevating both enzymes. Most notably, ALAT increased by 38% to 65.01 IU/L. In stark contrast, *Artemisia herba alba* extract at both doses did not induce liver injury. In the AHA-treated groups, ALAT levels remained within the normal range, Figure 6B,C.

#### 3.4.7. Renal Function Biomarkers (Urea and Creatinine)

Assessment of renal function revealed that blood urea nitrogen levels showed no significant changes across any experimental group, with all values ranging from 1.1 to 1.8 mg/L, Figure 6D. Creatinine levels, however, told a different story. The induction of arthritis impaired kidney function, increasing serum creatinine by 50% from 0.52 mg/L in normal rats to 0.78 mg/L. Indomethacin treatment only partially attenuated this increase, resulting in a still-elevated level of 0.65 mg/L. *Artemisia herba alba* extract, however, showed a renoprotective, dose-dependent normalization. The 250 mg/kg dose effectively brought creatinine down to 0.58 mg/L, a level not statistically different from normal, while the 500 mg/kg dose achieved a statistically significant protective effect, lowering it to 0.54 mg/L (*p* < 0.05) Figure 6E.

### 3.5. Hematological Parameters

**Figure 7:**
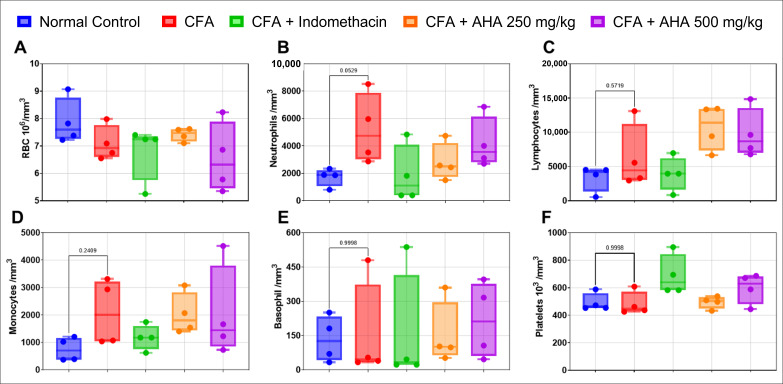
Mean hematological parameters are represented as follows: (**A**) for red blood cells (RBC), (**B**) for neutrophils, (**C**) for lymphocytes, (**D**) for monocytes, (**E**) for basophils, and (**F**) for platelets. Data are mean ± SEM of four rats. Data presented as min–max.

The induction of arthritis in rats via CFA injection did not yield statistically significant changes in hematological parameters in all arthritic groups. Despite this lack of statistical significance (*p* > 0.05), there was a noticeable trend towards increased neutrophils, lymphocytes, and monocytes observed in the arthritic rats compared to the normal control group, Figure 5. While these changes did not meet the threshold for statistical significance, they suggest a potential immune response triggered by the arthritis induction. In the treatment groups receiving indomethacin as well as *Artemisia herba alba* at 250 and 500 mg/kg, the hematological parameters likewise did not show any statistically significant differences when compared with the CFA group (*p* > 0.05). Although slight variations in red blood cells, platelets, and leukocyte subpopulations were observed across the treated groups, these fluctuations remained within a comparable range to the arthritic control and did not reach statistical significance (Figure 7).

### 3.6. Histopathological Change

**Figure 8:**
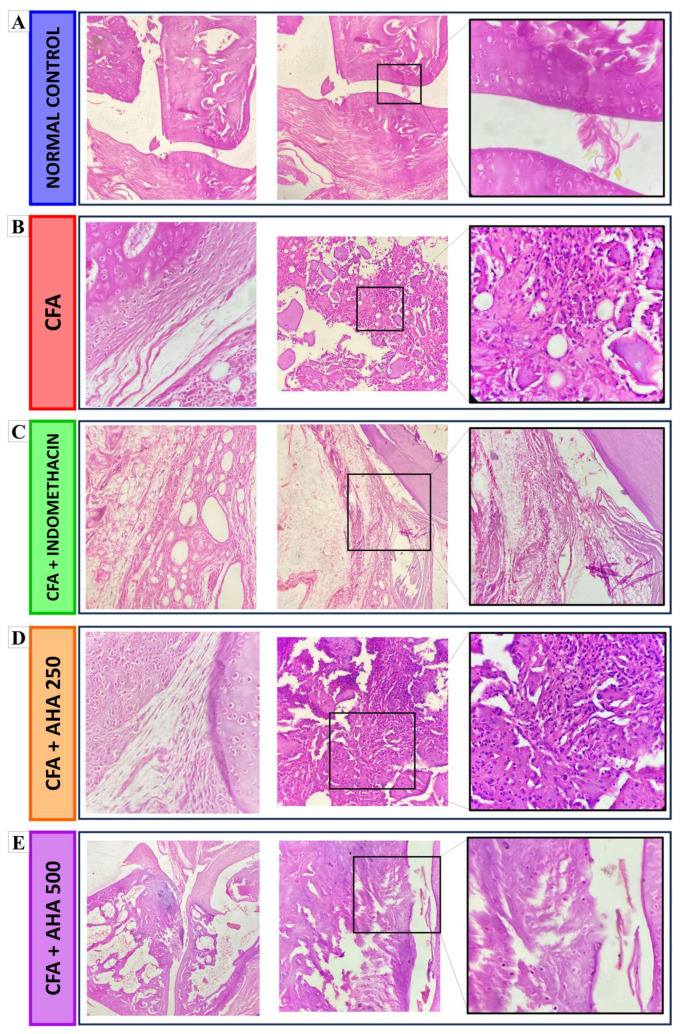
Effect of *Artemisia herba alba* (AHA) on the histopathology of bone in arthritic rats at 40×, 100×, 400× magnifications. The tissues were stained with H&E stains. Here: (**A**) normal control; (**B**) CFA-arthritic control; (**C**) rats treated with indomethacin at a dose of 3 mg/kg; (**D**) rats treated with AHA at a dose of 250 mg/kg; (**E**) rats treated with AHA at a dose of 500 mg/kg. The images are a representative sample of all the examined tissues.

Histopathological examination of hematoxylin and eosin-stained joint sections revealed marked differences between the experimental groups, which were further supported by semi-quantitative scoring of inflammatory cell infiltration and bone erosion (Figure 8). The normal control group showed a well-preserved articular architecture, with smooth joint surfaces, intact hyaline cartilage, and a regularly organized subchondral bone.

**Figure 9:**
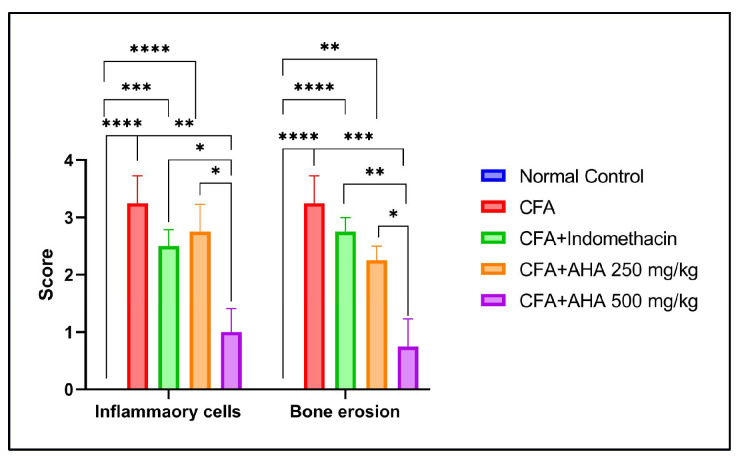
Semi-quantitative histopathological scoring of joint sections showing inflammatory cell infiltration and bone erosion in normal control, CFA, CFA + indomethacin, and CFA + *Artemisia herba alba* (AHA; 250 and 500 mg/kg)–treated rats. Data are mean ± SEM of four rats. Multiple comparisons between groups are represented. Statistical significance is indicated as * *p* < 0.05, ** *p* < 0.01, *** *p* < 0.001, and **** *p* < 0.0001.

In contrast, CFA-treated rats exhibited severe pathological alterations typical of inflammatory arthritis, including pronounced synovial hyperplasia, dense inflammatory cell infiltration, and pannus formation extending into the joint space. These changes were associated with marked cartilage disorganization and early erosion of the subchondral bone (Figure 8B). Accordingly, CFA induction resulted in a significant increase in histopathological scores for both inflammatory cells and bone erosion (3.25 ± 0.47) compared with the normal control group (*p* < 0.0001) Figure 9.

Treatment with indomethacin partially attenuated these pathological features, as evidenced by reduced synovial thickening and inflammatory infiltration, although residual inflammatory changes and incomplete structural restoration persisted (Figure 8C). Quantitatively, indomethacin did not significantly reduced inflammatory cell infiltration to 2.5 ± 0.28 (ns vs. CFA) and bone erosion to 2.75 ± 0.25 (ns vs. CFA), indicating a low protective effect Figure 9.

Rats treated with *Artemisia herba alba* at 250 mg/kg showed an intermediate histological improvement, characterized by partial preservation of cartilage structure, reduced pannus formation, and a moderate inflammatory infiltrate (Figure 8D). These observations were reflected by inflammatory cell and bone erosion scores of 2.75 ± 0.47 and 2.25 ± 0.25, respectively, which were not significantly lower than those of the CFA group, yet remained higher than the normal control values (*p* < 0.0001–0.01) and indomethacin-treated group (*p* < 0.05) regarding inflammatory cell infiltration, Figure 9.

In contrast, administration of *Artemisia herba alba* at 500 mg/kg resulted in a near-complete restoration of joint morphology, with well-preserved cartilage, minimal synovial cellularity, and an absence of evident pannus formation or bone erosion (Figure 8E). Consistently, this group displayed the lowest histopathological scores among treated animals, with inflammatory cells scored at 1 ± 0.4 (*p* < 0.01 vs. CFA) and bone erosion at 0.75 ± 0.47 (*p* < 0.001 vs. CFA), representing a highly significant reduction compared with the CFA group and a superior protective effect relative to both indomethacin (*p* < 0.05–*p* < 0.01) and the lower *Artemisia herba alba* dose (*p* < 0.05), Figure 9.

## 4. Discussion

The present study demonstrates that *Artemisia herba alba* extract confers significant therapeutic benefits in CFA-induced monoarthritis, as reflected across functional, biochemical, behavioral, and histopathological outcomes in both the main results and supplementary data. The higher dose (500 mg/kg) consistently produced the strongest effects, reducing paw swelling, improving locomotor activity, decreasing mechanical hypersensitivity, and protecting joint architecture. Importantly, these effects were achieved without evidence of hepatic or renal toxicity, in contrast to indomethacin, which induced oxidative stress and hepatocellular injury, see Figures S1 and S2. Taken together, these findings support the view that *Artemisia herba alba* exerts combined anti-inflammatory, analgesic, and antioxidant actions while maintaining a favorable safety profile.

Beyond the reduction in paw diameter, the anti-inflammatory activity of *Artemisia herba alba* was supported by clinical arthritis scoring, improvement in exploratory behavior, and attenuation of tactile allodynia. These complementary outcomes indicate that the extract influences both local inflammatory processes and functional manifestations of disease. In particular, the marked decrease in mechanical withdrawal responses in the 500 mg/kg group suggests a genuine antinociceptive effect rather than a secondary consequence of reduced edema [31]. This aligns with previous investigations of Artemisia species and phenolic-rich natural products, which have shown concomitant suppression of inflammation and pain perception in rodent arthritis and carrageenan models [32,33,34]. Compared with other plant-derived preparations evaluated in CFA-induced arthritis, *Artemisia herba alba* displayed a particularly robust effect on pain behavior and locomotor recovery, suggesting potential superiority in functional outcomes. It is also important to note that this experiment was conducted in adult rats over a 15-day period, during which CFA primarily blunted the normal physiological progression of body weight rather than inducing frank weight loss, while analgesic treatment restored expected weight gain in parallel with functional recovery.

The mechanisms underlying these effects may involve simultaneous modulation of inflammatory mediators and oxidative stress pathways [35]. *Artemisia herba alba* treatment restored total antioxidant capacity and prevented lipid peroxidation, whereas indomethacin markedly increased MDA levels and catalase activity, indicating compensatory oxidative stress. These results are consistent with the antioxidant and membrane-stabilizing properties of flavonoids and phenolic acids reported in Artemisia species and other medicinal plants used in inflammatory disorders [13,32,33,34,36,37,38]. In addition, the histopathological improvements observed in the *Artemisia herba alba*-treated groups correlate with these biochemical effects. Reduction in synovial hyperplasia, diminished inflammatory cell infiltration, and preservation of cartilage structure suggest that *Artemisia herba alba* effectively suppresses local inflammatory signaling within the joint microenvironment. These morphological changes are likely mediated through downregulation of pro-inflammatory cytokines such as TNF-α, IL-1β, and IL-6, which are known to drive pannus formation, cartilage degradation, and osteoclast activation in CFA-induced arthritis models [39]. Moreover, the observed preservation of subchondral bone and attenuation of pannus invasion in the higher-dose *Artemisia herba alba* group may reflect inhibition of osteoclastogenesis and matrix metalloproteinase activity, processes closely linked to oxidative stress and chronic inflammation [40]. The parallel reduction in lipid peroxidation, restoration of antioxidant defenses, and normalization of metabolic related markers, including SDH activity, further supports a mechanism whereby *Artemisia herba alba* mitigates oxidative damage to synovial and cartilage tissues, thereby stabilizing cellular membranes and preventing progressive tissue degeneration [41]. In contrast, while indomethacin reduces overt inflammation, the associated increase in MDA and catalase activity indicates a compensatory response to oxidative stress, which may explain the persistence of some histopathological alterations in the synovium and cartilage. Together, these findings suggest that the joint-protective effects of *Artemisia herba alba* result from a dual action, combining anti-inflammatory modulation with robust antioxidant activity, ultimately preserving tissue integrity and preventing the structural consequences of chronic arthritis. Importantly, the antiradical activity of bioactive compounds absorbed from *Artemisia herba alba* extract may further reinforce this protective effect, as evidenced by the enhancement of serum TAC activity Table S4. Collectively, these complementary mechanisms may act synergistically to mitigate ROS-mediated cartilage degradation and preserve joint structural integrity, although further targeted investigations and molecular analyses are warranted to substantiate these pathways.

A major strength of the present work lies in the comparative assessment with indomethacin. Although indomethacin reduced inflammation, it also induced hepatotoxic and oxidative alterations, as reflected by increased transaminases, elevated lipid peroxidation, and structural hepatic and renal lesions confirmed by histopathology, Figure S2.

These findings are consistent with well-documented NSAID-associated organ toxicity reported in preclinical and clinical studies [42,43]. In contrast, *Artemisia herba alba* maintained normal liver and kidney biochemical parameters, preserved organ index values, and produced no histological abnormalities, Figure S2. Collectively, this indicates that *Artemisia herba alba* provides therapeutic benefit without compromising systemic safety, which is particularly relevant in the context of chronic inflammatory conditions requiring prolonged pharmacological management.

The extract’s efficacy may also be interpreted in light of phytochemical studies reporting that *Artemisia herba alba* contains flavonoids, phenolic acids, and sesquiterpene lactones with anti-inflammatory and antioxidant potential [11,44]. Previous in vivo and in silico work has suggested interactions with cytokine, COX-2, and NF-κB–related pathways [11,12]. Although our results are compatible with these mechanisms, the present study did not directly quantify molecular markers, and such mechanistic links should therefore be regarded as interpretative rather than demonstrative. Future studies incorporating cytokine profiling and pathway-specific assays will be necessary to clarify these molecular contributions.

This study nonetheless presents several limitations. First, the number of animals per group was relatively small (n = 4), which may limit statistical power and increase biological variability. Second, only male rats were included, and potential sex-dependent responses cannot be excluded. Third, although CFA monoarthritis is a validated and widely used model, it does not fully reproduce the systemic and autoimmune features of human rheumatoid arthritis. Finally, mechanistic interpretations remain partly speculative due to the absence of direct inflammatory and oxidative marker quantification. Similarly, the identification of bone-remodeling cells in joint sections was based on histomorphological features under H&E staining, and we acknowledge that definitive discrimination between osteoclasts and osteoblasts would require targeted immunohistochemical confirmation. Furthermore, larger, mechanistically oriented, and longer-term studies are therefore warranted.

## 5. Conclusions

In conclusion, this study positions *Artemisia herba alba* as a promising natural alternative for arthritis management, combining efficacy with a superior safety profile compared to conventional NSAIDs. The maintenance of normal hematological parameters alongside its anti-inflammatory and antioxidant effects further underscores its potential as a well-tolerated therapeutic option. As a pioneering investigation in the CFA-induced arthritis model, these promising results establish a valuable foundation for future research. Future research should explore its mechanisms of action in greater detail, particularly its effects on specific inflammatory signaling pathways, and evaluate its potential in clinical settings. Additionally, investigations into optimized formulations or synergistic combinations with existing therapies could further enhance its therapeutic utility.

## Figures and Tables

**Table 1:** Macroscopic arthritic scoring system (0–5) for CFA-induced arthritis in rats.

Score	Description
0	No observable changes; normal joint
1	Slight swelling or redness
2	Mild swelling and erythema; slight restriction of movement
3	Moderate swelling and redness; joint visibly inflamed, movement impaired
4	Severe swelling and erythema; limited movement
5	Immobility of the limb; severe arthritis

## Data Availability

The original contributions presented in this study are included in the article/Supplementary Materials. Further inquiries can be directed to the corresponding author.

## Supplementary Materials

The following supporting information can be downloaded at: https://www.mdpi.com/article/10.3390/antiox15020190/s1, Figure S1: The kidney and liver indexes. Data are mean ± SEM of four rats. ns non-significant, * *p* < 0.05, and **** *p* < 0.0001 versus arthritic control group and #### *p* < 0.0001 versus indomethacin group; Figure S2: Effect of *Artemisia herba alba* (AHA) and indomethacin on the histopathology of kidneys and liver in arthritic rats at 10× magnification. The tissues were stained with H&E stains. Here: A: arthritic control; B: negative control; C: rats treated with indomethacin at a dose of 3 mg/kg; D: rats treated with AHA at a dose of 250 mg/kg; E: rats treated with AHA at a dose of 250 mg/kg. Table S1. Paw diameter and body weight of rats were taken one day after CFA injection followed by a measurement every 5 days. Data are mean ± SEM of four rats. ns: non-significant, ** *p* < 0.01, *** *p* < 0.001, **** *p* < 0.0001 versus CFA group, and # *p* < 0.05, ## *p* < 0.01 versus CFA + indomethacin group, and § *p* < 0.05, §§ *p* < 0.01, §§§§ *p* < 0.0001 versus normal control group. Table S2. % of inhibition of induced oedema in comparison to CFA groups. Table S3: Mechanical withdrawal response percentages (%) for von Frey filament tests (1 g, 5 g, 10 g, 15 g) across experimental groups (normal control, CFA arthritic control, CFA + indomethacin, CFA + *Artemisia herba alba* (AHA) at 250 mg/kg, and 500 mg/kg) at days 0, 1, and 15 post-CFA in-duction. Data are % mean ± SEM of four rats. Table S4. Effects of *Artemisia herba alba* (AHA) extract on oxidative stress, antioxidant defense, lipid peroxidation, and hepatorenal biochemical parameters in CFA-induced arthritic rats. The table summarizes total antioxidant capacity (TAC; absorbance at 695 nm), DPPH free radical scavenging activity (% inhibition), total protein content (µg/mL), catalase (CAT) activity (nmol/min/mg protein), succinate dehydrogenase (SDH) activity (nmol/min/mg protein), malondialdehyde (MDA) levels (µmol/L), aspartate aminotransferase (ASAT) activity (U/L), alanine aminotransferase (ALAT) activity (U/L), serum urea (mg/L), and serum creatinine (mg/L). Data are expressed as mean ± SEM.

## Abbreviations

The following abbreviations are used in this manuscript:

CFA	Complete Freund’s adjuvant
AHA	*Artemisia herba alba*
NSAIDs	Non-steroidal anti-inflammatory drugs
COX	Cyclooxygenase
NF-κB	Nuclear Factor kappa-light-chain-enhancer of activated B cells
RBC	Red blood cells
TAC	Total antioxidant capacity
MDA	Malondialdehyde
ASAT	Aspartate aminotransferase
ALAT	Alanine aminotransferase
SDH	Succinate dehydrogenase
MMP	Matrix metalloproteinases
ROS	Reactive oxygen species
EDTA	Ethylenediaminetetraacetic acid
H&E	Hematoxylin and eosin
CAT	Catalase
DCIP	Dichlorophenolindophenol
TBARS	Thiobarbituric acid reactive substances
OF	Open field
CC	Chondrocytes
PBS	Phosphate-buffered saline
DPPH^•^	2,2-Diphenyl-1-picrylhydrazyl
