# Supplementary material for: Artemisia herba alba Outperforms Indomethacin with Multitarget Efficacy and Safety in CFA Arthritic Model
Source: Antioxidants (Basel). 2026 Feb 2;15(2):190. doi: 10.3390/antiox15020190 (PMC12938658; doi:10.3390/antiox15020190)
Supplement: Supplementary file 1 [file antioxidants-15-00190-s001.zip › antioxidants-4061236-supplementary.pdf]

Supplementary Materials:

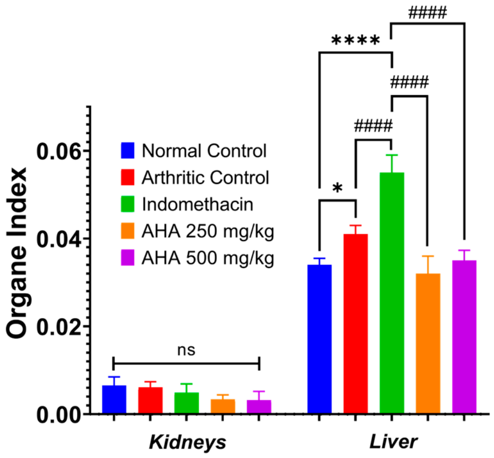

Figure S1. The kidney and liver indexes. Data are mean ± SEM of four rats. ns non-significant, *$p < 0.05$, and****$p < 0.0001$ versus arthritic control group and ####$p < 0.0001$ versus indomethacin group.

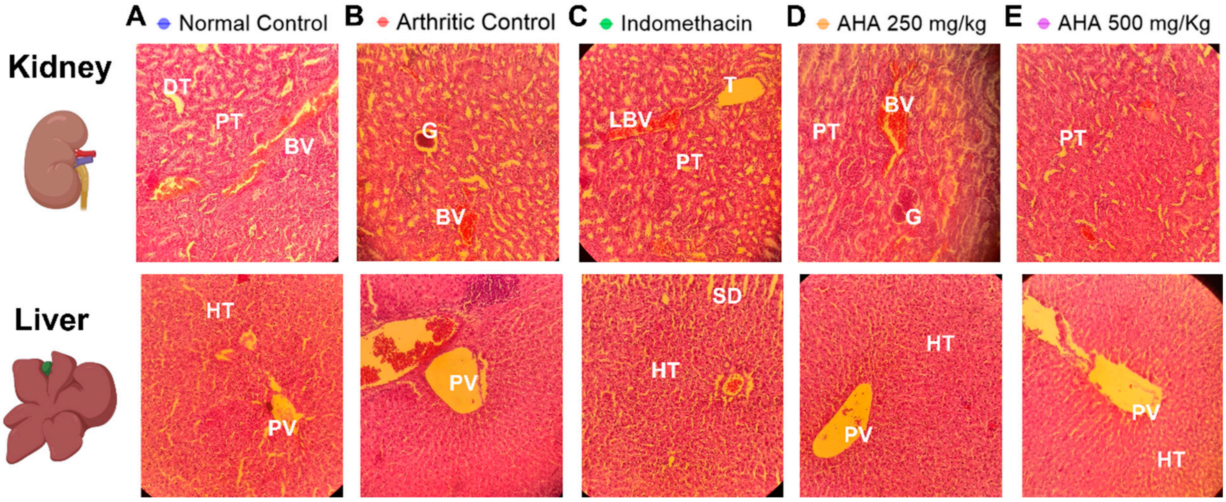

Figure S2. Effect of *Artemisia herba alba* (AHA) and indomethacin on the histopathology of kidneys and liver in arthritic rats at 20 × magnification. The tissues were stained with H&E stains. Here: **A:** arthritic control; **B:** negative control; **C:** rats treated with indomethacin at a dose of 3 mg/kg; **D:** rats treated with AHA at a dose of 250 mg/kg; **E:** rats treated with AHA at a dose of 500 mg/kg. DT: Distal convoluted Tubule, PT: Proximal convoluted Tubule, LBV: Large thick wall congested Blood Vessel, BV: Blood Vessel, G: Glomeruli, T: destructed Tubules, HT: Hepatocyte, PV: Portal Vein, SD: Structural Damage.

Table S1. Paw diameter and body weight of rats were taken one day after CFA injection followed by a measurement every 5 days. Data are mean ± SEM of four rats. ns: non-significant, **$p < 0.01$, ***$p < 0.001$, ****$p < 0.0001$ versus CFA group, and #$p < 0.05$, ##$p < 0.01$ versus CFA+indomethacin group, and § $p < 0.05$, §§ $p < 0.01$, §§§§ $p < 0.0001$ versus normal control group

	Paw diameter of the rats in mm Mean ± SEM (change%)				
	Day 0	Day 1	Day 5	Day 10	Day 15
**Normal Control**	4,57±0.04 ^{ns,ns}	4,55±0.05 $^{**,\#}$	4,55±0.05 $^{****,\#\#\#}$	4,575±0.04 $^{****,\#}$	4,55±0.05 $^{****,\#}$

		(-0.43%)	(-0.43%)	(0%)	(-0. 43%)
**CFA**	4,75±0.13 ^{ns}	5,525±0.1 ^{ns} (16.31%)	5,5±0.04 ^{ns} (15.78%)	5,675±0.04 ^{ns} (19.47%)	5,675±0.06 $^{\#\#, §§§§}$ (19.47%)
**CFA+Indometha cin**	4,7±0.04 ^{ns}	5,1±0.09 ^{ns} (8.5%)	5,25±0.06 ^{ns} (11.7%)	5,225±0.11 ^{ns} (11.17%)	5,075±0.08 $^{**, §§}$ (7.97%)
**CFA+AHA 250 mg/Kg**	4,72±0.04 ^{ns,ns}	4,925±0.02 ^{ns,ns} (4.23%)	5,175±0.02 ^{**,ns} (9.52%)	5,175±0.02 ^{**,ns} (9.52%)	4,9±0.07 $^{****,ns, §}$ (3.7%)
**CFA+AHA 500 mg/Kg**	4,6±0.05 ^{ns,ns}	4,9±0.04 ^{ns,ns} (6.52%)	5,025±0.04 ^{***,ns} (9.23%)	4,850±0.06 ^{***,ns} (5.43%)	4,650±0.02 $^{***,\#,ns}$ (1.08%)

**Groups**	**Body weight of rats in g Mean ± SEM**				
	**Day 0**	**Day 1**	**Day 5**	**Day 10**	**Day 15**
**Normal Control**	185,75±5.8	184,250±4.71	190±1.68	197,5±4.33	205±6.78
**CFA**	185,75±14.49	186,25±2.49	178,5±4.44	186±4.24	199±6.37
**CFA+Indometha cin**	182,25±4.46	182,75±5.08	179,5±4.17	181±3.89	189,5±3.7
**CFA+AHA 500 mg/Kg**	182±2.55	189,75±2.49	189,75±1.49	197,75±2.17	210,5±2.9
**CFA+AHA 250 mg/Kg**	185,75±4.4	184,5±5.18	194±3.91	201±2.85	214±1.29

Table S2. % of inhibition of induced oedema in comparaison to CFA groups

	**Day 1**	**Day 5**	**Day 10**	**Day 15**
**CFA+Indometha cin**	48,39 %	27,27 %	56,52 %	75,00 %
**CFA+AHA 250 mg/Kg**	73,55 %	33,33 %	66,67 %	88,46 %
**CFA+AHA 500 mg/Kg**	61,29 %	58,33 %	108,70 %	134,62 %

Table S3: Mechanical withdrawal response percentages (%) for von Frey filament tests (1g, 5g, 10g, 15g) across experimental groups (Normal Control, CFA Arthritic Control, CFA+Indomethacin, CFA+*Artemisia herba alba* (AHA) at 250 mg/kg, and 500 mg/kg) at days 0, 1, and 15 post-CFA in-duction. Data are % mean ± SEM of four rats.

Day 0 : Withdrawal response % (Mean ± SEM)					
**Poids (g)**	**Normal Control**	**CFA**	**CFA +Indomethacin**	**CFA +AHA 250 mg/kg**	**CFA +AHA 500 mg/kg**
**1 g**	5 ± 2.2	10 ± 3.2	5 ± 2.2	5 ± 2.2	5 ± 2.2
**5 g**	15 ± 4.1	20 ± 5.0	15 ± 4.1	15 ± 4.1	10 ± 3.2
**10 g**	0 ± 0.0	5 ± 2.2	0 ± 0.0	5 ± 2.2	0 ± 0.0
**15 g**	0 ± 0.0	0 ± 0.0	0 ± 0.0	0 ± 0.0	0 ± 0.0
Day 1 : Withdrawal response % (Mean ± SEM)					
**Poids (g)**	**Normal Control**	**CFA**	**CFA +Indomethacin**	**CFA +AHA 250 mg/kg**	**CFA +AHA 500 mg/kg**
1 g	5 ± 2.2	90 ± 4.5	50 ± 7.1	60 ± 6.7	40 ± 6.1
5 g	5 ± 2.2	80 ± 6.1	45 ± 6.8	55 ± 7.1	20 ± 4.5
10 g	0 ± 0.0	60 ± 6.7	25 ± 4.8	35 ± 5.7	10 ± 3.2
15 g	0 ± 0.0	25 ± 4.8	10 ± 3.2	15 ± 4.1	5 ± 2.2
Day 15 : Withdrawal response % (Mean ± SEM)					
**Poids (g)**	**Normal Control**	**CFA**	**CFA +Indomethacin**	**CFA +AHA 250 mg/kg**	**CFA +AHA 500 mg/kg**
1 g	5 ± 2.2	70 ± 6.1	30 ± 5.0	40 ± 6.1	10 ± 3.2
5 g	4 ± 2.2	60 ± 6.7	25 ± 4.8	35 ± 5.7	10 ± 3.2
10 g	0 ± 0.0	30 ± 5.0	10 ± 3.2	15 ± 4.1	5 ± 2.2
15 g	0 ± 0.0	10 ± 3.2	5 ± 2.2	5 ± 2.2	0 ± 0.0

Table S4. Effects of *Artemisia herba alba* (AHA) extract on oxidative stress, antioxidant defense, lipid peroxidation, and hepatorenal biochemical parameters in CFA-induced arthritic rats. The table summarizes total antioxidant capacity (TAC; absorbance at 695 nm), DPPH free radical scavenging activity (% inhibition), total protein content (µg/mL), catalase (CAT) activity (nmol/min/mg protein), succinate dehydrogenase (SDH) activity (nmol/min/mg protein), malondialdehyde (MDA) levels (µmol/L), aspartate aminotransferase (ASAT) activity (U/L), alanine aminotransferase (ALAT) activity (U/L), serum urea (mg/L), and serum creatinine (mg/L). Data are expressed as mean ± SEM.

	**Normal Control**	**CFA**	**CFA +Indomethacin**	**CFA +*AHA* 250 mg/kg**	**CFA +*AHA* 500 mg/kg**
TAC (nm)	*0,638±0,05*	*0,491±0.012*	*0,364±0,09*	*0,642±0.024*	*0,741±0.016*
DPPH (scavenging %)	*14,643±1,315*	*12,698±2,025*	*10,25±2,096*	*20,517±8,196*	*20,907±10,494*

Protein content (µg/ml)	*2428,947±136,395*	*3491,227±70,177*	*3469,3±22,81*	*3166,667±103,419*	*2550,88±15,294*
CAT (nmol/min/mg of protein)	*150,84±110,98*	*156,96±60,26*	*661,35±63,3*	*124,49±54,71*	*114,83±58,25*
SDH (nmol/min/mg of protein)	*347,25±28,29*	*306,08±95,5*	*218,46±54,12*	*327,44±72,62*	*298,5±16,45*
MDA (µmol/L)	*2,15±0,04*	*1,10±0,32*	*4,25±0,41*	*1,13±0,19*	*1,52±0,15*
ASAT (IU/L)	*169.5 ± 0.04*	*160.3 ± 12.7*	*185.6 ± 9.8*	*175.2 ± 8.4*	*172.8 ± 10.1*
ALAT (IU/L)	*45.2 ± 3.5*	*47.1 ± 2.2*	*65.01 ± 1.5*	*42.9 ± 4.2*	*46.6 ± 0.3*
Urea (mg/L)	*1.7 ± 2.8*	*1.3 ± 3.6*	*1.5 ± 3.2*	*1.1 ± 2.9*	*1.8 ± 3.1*
Creatinine (mg/L)	*0.52 ± 0.05*	*0.78 ± 0.07*	*0.65 ± 0.06*	*0.58 ± 0.05*	*0.54 ± 0.04*
